# Electrophysiological characterization of human rectal afferents

**DOI:** 10.1152/ajpgi.00153.2016

**Published:** 2016-10-27

**Authors:** Kheng-Seong Ng, Simon J. Brookes, Noemi A. Montes-Adrian, David A. Mahns, Marc A. Gladman

**Affiliations:** ^1^Academic Colorectal Unit, Sydney Medical School, Concord, University of Sydney, Sydney, Australia;; ^2^Enteric Neuroscience and Gastrointestinal Research Group, ANZAC Research Institute, University of Sydney, Sydney, Australia;; ^3^Discipline of Human Physiology, FMST, School of Medicine, Flinders University, Adelaide, Australia; and; ^4^Department of Integrative Physiology, School of Medicine, Western Sydney University, Sydney, Australia

**Keywords:** electrophysiology, human, rectal afferents

## Abstract

It is presumed that extrinsic afferent nerves link the rectum to the central nervous system. However, the anatomical/functional existence of such nerves has never previously been demonstrated in humans. Therefore, we aimed to identify and make electrophysiological recordings in vitro from extrinsic afferents, comparing human rectum to colon. Sections of normal rectum and colon were procured from anterior resection and right hemicolectomy specimens, respectively. Sections were pinned and extrinsic nerves dissected. Extracellular visceral afferent nerve activity was recorded. Neuronal responses to chemical [capsaicin and “inflammatory soup” (IS)] and mechanical (Von Frey probing) stimuli were recorded and quantified as peak firing rate (range) in 1-s intervals. Twenty-eight separate nerve trunks from eight rectums were studied. Of these, spontaneous multiunit afferent activity was recorded in 24 nerves. Peak firing rates increased significantly following capsaicin [median 6 (range 3–25) spikes/s vs. 2 (1–4), *P* < 0.001] and IS [median 5 (range 2–18) spikes/s vs. 2 (1–4), *P* < 0.001]. Mechanosensitive “hot spots” were identified in 16 nerves [median threshold 2.0 *g* (range 1.4–6.0 *g*)]. In eight of these, the threshold decreased after IS [1.0 g (0.4–1.4 *g*)]. By comparison, spontaneous activity was recorded in only 3/30 nerves studied from 10 colons, and only one hot spot (threshold 60 *g*) was identified. This study confirms the anatomical/functional existence of extrinsic rectal afferent nerves and characterizes their chemo- and mechanosensitivity for the first time in humans. They have different electrophysiological properties to colonic afferents and warrant further investigation in disease states.

## Abstract (precis)

*This study confirms the existence of extrinsic nerves supplying the human rectum for the first time and demonstrates differences in the sensory innervation between the rectum and colon with rectal afferents being more mechanically and chemically sensitive than colonic afferents. As sensitization of gut afferent pathways appears important in the development of chronic pain in patients with functional bowel disorders, this in vitro model will allow evaluation of potential therapeutic agents on human visceral afferents*.

## NEW & NOTEWORTHY

*This study confirms the existence of extrinsic nerves supplying the human rectum for the first time and demonstrates differences in the sensory innervation between the rectum and colon with rectal afferents being more mechanically and chemically sensitive than colonic afferents. As sensitization of gut afferent pathways appears important in the development of chronic pain in patients with functional bowel disorders, this in vitro model will allow evaluation of potential therapeutic agents on human visceral afferents*.

the importance of normal function of the human rectum is often only truly appreciated when the impact of symptoms resulting from dysfunction of this highly specialized, terminal segment of the gastrointestinal tract is considered. Abnormal sensation appears to be particularly relevant in the development of rectal dysfunction and is most obvious, in the clinical setting, in the context of inflammatory bowel disease in which heightened sensation (rectal hypersensitivity) (18) occurs following mucosal and/or transmural inflammation. Inflammatory sensitization often manifests as an irrepressible urge to defecate, leading to severe fecal urgency, frequently associated with episodes of incontinence (18). Furthermore, stimulation of nociceptive afferents may contribute to the development of pain (16). However, abnormal rectal sensation can affect function in the absence of organic disease, manifesting as disorder(s) of evacuation and/or storage of feces. Rectal hyposensitivity (22, 23) and hypersensitivity (11) are prevalent and are associated with chronic constipation and/or fecal incontinence. Abnormal rectal sensitivity has also been proposed as a biomarker of irritable bowel syndrome (36, 38). Collectively, these functional bowel disorders account for up to 41% of diagnoses in specialty practices (13) and have been estimated to affect over one-third of the Western adult population in community studies (28). Additionally, a significant proportion of patients develop hindgut dysfunction following complete or partial excision of the rectum (proctectomy), termed “anterior resection syndrome” (42), which has been in part attributed to disturbances of (neo)rectal sensory function (9).

Despite the functional and clinical importance of the rectum, detailed understanding of normal rectal physiology, particularly its sensorimotor function, remains incompletely understood, and thus the ability to manage these clinical conditions remains suboptimal. The rectum provides graded sensory information to the brain, reflecting varying degrees of distension (18, 37, 45), pointing to the existence of a specialized extrinsic afferent pathway in humans. However, early histopathological studies failed to identify specific receptors in the human rectum (17), leading to the belief that it lacked specialized sensory receptors and that rectal sensation was mediated solely by nerve endings and receptors in adjacent pelvic structures and pelvic floor musculature (39), particularly because patients still experience sensations of fullness and impending defecation following excision of the entire rectum (complete proctectomy) and coloanal anastomosis (29, 44). However, it has come to be appreciated that these neorectal sensations are not typical of normal rectal filling (44). Thus awareness of filling is preserved after the anorectum is transposed on its neurovascular pedicle to the anterior abdominal wall (46) (despite being anatomically distinct and remote to the pelvis), strongly suggesting that the rectum (and/or its mesentery) itself is innervated by extrinsic afferent nerves.

The anorectum is innervated by somatic and autonomic pathways (8), and animal studies have demonstrated that the hindgut is innervated via two afferent populations that arise from different levels of the spinal cord (thoracolumbar and lumbosacral) (14). However, studies have shown that the rectum receives different classes of extrinsic sensory neurons to the colon (6), and this includes specialized low-threshold mechanoreceptors (32), several classes of high-threshold nerve endings, and mucosal receptors (1, 6). Specialized rectal afferent nerve endings [rectal intraganglionic laminar endings (rIGLEs)] are the sites of mechanotransduction of low-threshold mechanoreceptors in the guinea pig rectum (32). Comparable studies of human rectal preparations have not been carried out although electrophysiological studies have recorded sensory nerve activity from the human colon and appendix in vitro (35). Additionally, two very recent studies have utilized ex vivo electrophysiological recording techniques to identify functionally distinct subpopulations of human visceral afferents from different parts of the lower gastrointestinal tract (including the ileum). However, much of the focus was again placed on colonic tissue (33, 48). Therefore, this study aimed to identify and record visceral afferent activity from extrinsic nerves supplying the human rectum in vitro and to characterize their response to mechanical and chemical stimulation, making comparisons between rectal and colonic afferents.

## MATERIALS AND METHODS

### 

#### Subjects.

This study was approved by the Sydney Local Health District Human Research Ethics Committee (LNR/12/CRGH/41). Fresh, noninflamed rectal and colonic tissues were procured from specimens of consecutive patients undergoing elective anterior resection (proctectomy) or right hemicolectomy surgery for malignant or benign pathology between July 2013 and November 2013. Exclusion criteria included patients *1*) younger than 18 yr old, *2*) who had the possibility of a functional disorder (e.g., slow transit constipation, rectal prolapse), and *3*) previously diagnosed with inflammatory bowel disease.

#### Tissue procurement.

**Fig. 1.**
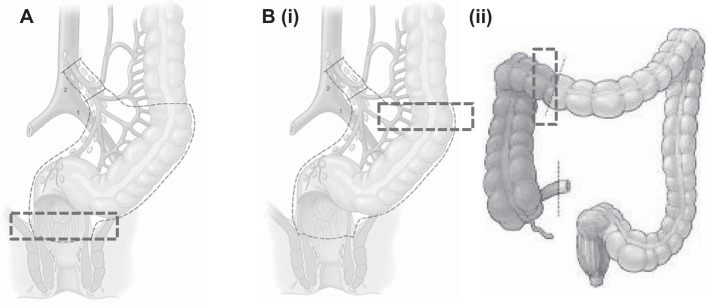
Diagram showing tissues procured for the study. *A*: rectal tissue, acquired from the distal (aboral) end of an anterior resection specimen. *B*: colonic tissue, acquired from the proximal (oral) end of an anterior resection specimen (*i*) or the distal (aboral) end of a right hemicolectomy specimen (*ii*).

Specimens were resected according to standard surgical protocol. Immediately following specimen removal, full-thickness circumferential sections (1- to 2-cm width) of rectal or colonic tissue with attached mesentery (>3 cm) were isolated from the resection margins. Specifically, rectal tissue was acquired from the distal (aboral) portion of an anterior resection (proctectomy) specimen and colonic tissue from either the proximal (oral) end of anterior resection (proctectomy) specimen or the distal (aboral) end of a right hemicolectomy specimen (Fig. 1). All tissue samples obtained were macroscopically free of disease. If scattered diverticula were evident in left-sided specimens, these were avoided in specimen procurement. Tissues were then immediately immersed in Krebs solution (in mmol/l: 118.00 NaCl, 4.75 KCl, 1.00 NaH_2_PO_4_, 25.00 NaHCO_3_, 1.20 MgSO_4_, 2.50 CaCl_2_, and 11.00 glucose; Sigma Aldrich, NSW, Australia) at room temperature to minimize ischemia time; the Krebs solution had been preoxygenated by bubbling with carbogen (95% O_2_-5% CO_2_; BOC, NSW, Australia). Tissues were then transported directly to the laboratory.

#### Tissue preparation.

**Fig. 2.**
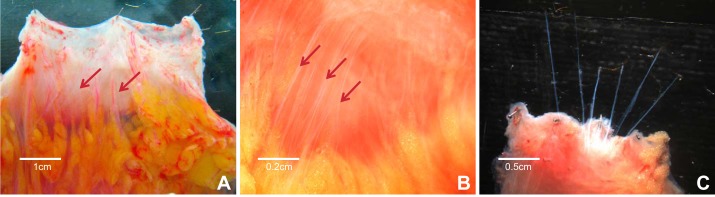
*A*: tissue pinned serosa side up (×1 magnification). Arrows indicate mesenteric nerves running in neurovascular bundles. *B*: view under a dissecting microscope (×2 magnification). Arrows indicate mesenteric nerves. *C*: nerve trunks dissected free from surrounding connective tissue.

On arrival in the laboratory, tissues were transferred to a Petri dish lined with Sylgard (Dow Corning, Midland, MI) and oriented with respect to its mesentery. Epiploic fat was excised from each specimen and the tissue opened by incising longitudinally along its antimesenteric border such that the mesentery lay along one edge of the open preparation. The preparation was pinned flat (mucosa side up) using stainless steel pins (200-μm diameter; Australian Entomological Supplies, NSW, Australia). Under a dissecting microscope (Olympus, Victoria, Australia), the mucosa was removed by sharp dissection from its submucosal attachments, and then the tissue was turned over and repinned serosa upward (Fig. 2).

#### Mesenteric nerve dissection.

Using a dissecting microscope, fine paravascular extrinsic nerve trunks were dissected free from surrounding connective tissue (Fig. 2). Between 6 and 10 nerve trunks were isolated for a length of 10–20 mm, then pinned using gold-plated tungsten pins (50-μm diameter; Goodfellow, Coraopolis, PA). Finally, the bowel wall was cut down to dimensions of 20 mm × 20 mm according to the distribution of nerves isolated. During the entire dissection, the Krebs solution contained within the Petri dish was regularly exchanged for fresh oxygenated solution, approximately every 10 min.

#### Electrophysiology.

**Fig. 3.**
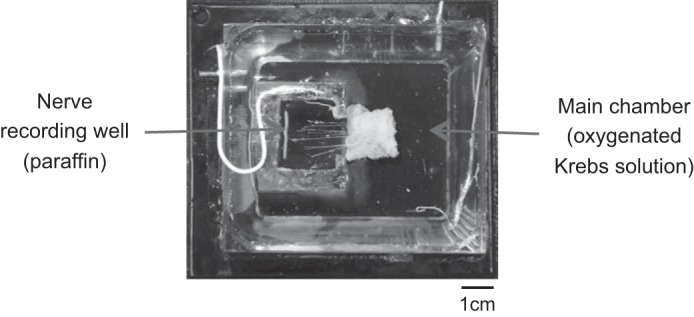
Specialized tissue chamber containing a separate paraffin-filled nerve recording well that could be isolated from the main chamber.

The entire preparation was transferred to a Sylgard-lined organ bath containing a nerve-recording chamber (Fig. 3), machined from clear acrylic. The organ bath was continually superfused at 5 ml/min with oxygenated Krebs solution. The preparation was pinned with steel pins (200 μm) along three sides, with the free edge connected to an array of hooks, attached to a pulley-weight system applying a 20 mN load. The dissected mesenteric nerves were led into the recording chamber and the ends fixed with 50-μm tungsten pins. A thin strand of connective tissue was also led into the chamber and attached to a reference electrode. The recording chamber was then sealed with a coverslip sealed with silicon grease (Ajax, Taren Point, Australia). Krebs solution was then removed from the recording chamber and replaced with paraffin oil. The entire organ bath was then placed on a water-perfused heating plate to achieve a temperature of 34°C within a Faraday cage.

The mesenteric nerves were recorded via a silver hook electrode attached to a low-noise AC-coupled differential amplifier (gain × 10,000), band pass filtered from 0.3–10.0 kHz (World Precision Instruments, Sarasota, FL), and sampled at 20 kHz via a CED 1401 interface (Cambridge Electronic Design, Cambridge, United Kingdom). Data were stored on an IBM-compatible personal computer for offline analysis. The amplified signal was also used for online audio monitoring.

#### Assessment of tissue mechano- and chemosensitivity.

The following features were recorded in a standardized protocol: *1*) spontaneous afferent nerve activity (for 5 min), *2*) response(s) to mechanical stimulation, *3*) response(s) to chemical stimulation, and *4*) response(s) to mechanical stimulation post chemical stimulation. Mechanical stimulation was assessed by systematically probing the tissue at 5-mm intervals with von Frey hairs of varying force (0.05–60.00 *g*). A “descending method of limits” was employed, beginning with 60.0 *g* of force and progressively decreasing in increments as defined by the Semmes-Weinstein monofilament set (30). Von Frey hairs were applied to the preparations in a grid extending over the full width and length of the preparations. Small (typically <200 μm in diameter), responsive sites, where firing was evoked, were identified as “hot spots,” and their position was marked on the tissue with fine carbon particles attached to the tip of the von Frey hair. The threshold for each hot spot was determined.

Responses to chemical stimulation were assessed by superfusing the main chamber with *1*) a hyperkalemic Krebs solution ([K^+^] = 6 mM) with an “inflammatory soup” [IS; bradykinin, serotonin, and histamine from frozen aqueous aliquots and prostaglandin E_2_ dissolved in dimethylsulfoxide (19) all at 10 μM final concentration] and *2*) Krebs solution containing capsaicin (10 μM from ethanolic stock solution) applied at the end of the study protocol to avoid desensitization. The preparation was systematically reprobed with von Frey hairs following exposure to IS to assess sensitization of receptors. Specifically, hot spots identified on initial probing (as marked by carbon particles) were reassessed, and changes in threshold and emergence of new hot spots were determined. This protocol was employed for each nerve trunk recorded. Between recordings, the chamber was superfused with oxygenated Krebs for at least 5 min to flush away any residual chemical stimulants.

#### Nerve activity analyses.

Action potentials were analyzed offline using Spike2 software (version 7; CED, Cambridge, United Kingdom). Actions potentials were discriminated by waveform characteristics; the threshold was set to the smallest identifiable spike. Nerve activity was expressed as a rate histogram with 1-s bin widths or as instantaneous frequency plots. Firing was analyzed according to *1*) peak firing rate, defined as the maximum number of action potentials in a 1-s bin, and *2*) mean firing rate, defined as the average number of action potentials per second averaged over 10 s. Paired comparisons of these firing rates were compared with baseline rates using the Wilcoxon signed-rank test with significance set at *P* < 0.05.

Firing was also compared between rectum and colon; to minimize bias, discrimination of action potentials and calculation of firing rates were performed by an observer (K.-S. Ng) blinded to the tissue type or treatment. Discrimination parameters were then cross checked by an independent reviewer (D. Mahns), who was similarly blinded to the tissue type and treatment.

#### Electrical stimulation.

In preparations in which there was neither spontaneous firing nor mechanically induced firing, focal electrical stimulation was used to confirm nerve integrity from evoked compound action potentials with appropriate latencies; this check was performed for six colonic nerves (randomly selected) in which nerve activity was not initially recorded. A round-tipped concentric electrode (external diameter 0.550 mm, internal diameter 0.125 mm; FHC, Bowdoin, ME; 0.5-ms pulse duration, 0.3 Hz, up to 10.0 mA) was micromanipulated in light contact with the tissue. A stimulus intensity of 10 mA was used to excite afferent endings. The electrode was moved systematically (2- to 3-mm steps) across the preparation until action potentials were recorded. Then electrode position was adjusted to pinpoint the site of maximum activation. Conduction velocity was calculated from the straight-line distance between the stimulating cathode and the recording site, divided by the latency of the compound action potential. A conduction velocity of <2 m/s was considered to reflect activation of afferent C fibers.

## RESULTS

### 

#### Description of tissues procured.

Colonic and rectal tissues were available from consecutive resections, as no patients declined participation in the study. All tissues appeared macroscopically normal on arrival in the laboratory, evidenced by their color and the spontaneous contractility. Overall, 8 rectums and 10 colons were procured for the study, from which 28 rectal and 30 colonic nerves were studied, respectively. The patients from whom rectal and colonic tissues were procured were comparable for age [rectum: median 67 yr (range 38–82 yr); colon: 73 yr (57–82 yr), *P* = 0.244] and sex (rectum: 6 males; colon: 7 males, *P* = 0.618) (Table 1).

**Table 1.** Clinicopathological features of patients studied, divided into those from whom rectal or colonic tissues were acquired

Rectum (*n* = 8)	
Age, median (range)	67 yr (38–82)
Male, *n* (%)	6 (75%)
Pathology	
Cancer, *n* (%)	7 (88%)
Diverticular disease, *n* (%)	1 (12%)
Distance from anal verge	
11–15 cm (upper rectum)	5 (63%)
6–10 cm (mid rectum)	1 (12%)
1–5 cm (low rectum)	2 (25%)
Neoadjuvant radiotherapy, *n* (%)	2 (25%)
Colon (*n* = 10)	
Age, median (range)	73 yr (57–82)
Male (number, %)	7 (70%)
Pathology	
Cancer, *n* (%)	10 (100%)
Tissue type	
Transverse colon, *n* (%)	5 (50%)
Descending colon, *n* (%)	5 (50%)

Of rectal samples (*n* = 8), seven patients underwent surgery for rectal or sigmoid adenocarcinoma and one for diverticular disease. All colonic samples (*n* = 10) were from patients undergoing surgery for adenocarcinoma. The distance of rectal samples from the anal verge was variable, as dictated by the surgical extent of the resection. Five samples were obtained from the upper rectum (11–15 cm from the anal verge), one from the mid rectum (6–10 cm), and two from the low rectum (1–5 cm). Of the 10 colonic samples procured, five were from the proximal transverse colon at the aboral end of right hemicolectomy specimens, and five were from the distal descending colon at the oral end of anterior resection (proctectomy) specimens. Two (of 8) patients from whom rectum was procured received neoadjuvant radiotherapy (Table 1). Although no patients had any formal documented neurological conditions, two rectal and two colonic samples were taken from patients with type II diabetes mellitus without associated neurological complications.

#### Spontaneous nerve activity.

**Fig. 4.**
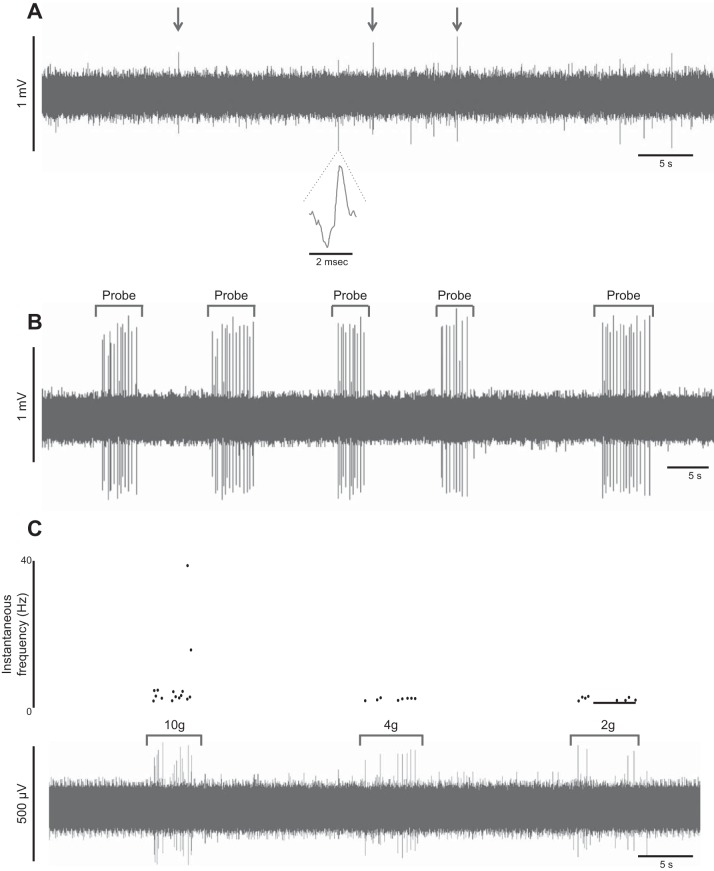
*A*: typical trace of spontaneous nerve activity (horizontal axis: time, s; vertical axis: voltage, mV), recorded from a rectal nerve (patient: 56-yr-old male). The specimen was of rectal tissue (8 cm from anal verge) acquired from anterior resection performed for rectosigmoid adenocarcinoma. Arrows indicate spontaneous nerve action potentials. The dashed lines show 1 of these action potentials at a faster time base, with a typical biphasic waveform. *B*: typical nerve response to repeated mechanical probing with a 4.0 *g* von Frey hair applied to a single marked hot spot on the preparation. Application is indicated by the interval bars. *C*: hot spot responses to a decreasing series of mechanical stimuli applied by von Frey hairs of 3 different stiffnesses. Firing rates decreased as probing force was reduced, confirmed on corresponding plot of instantaneous frequency (Hz), which could be used to identify the threshold according to the decreasing method of limits.

A typical trace of spontaneous activity is shown in Fig. 4*A*, with characteristic irregular spontaneous firing, which always persisted throughout the initial 5 min of recording, although occasional periods of quiescence without neuronal activity for up to 30 s were occasionally observed. Spontaneous firing was recorded in 24 of the 28 rectal nerves studied, with nerve activity recorded in nerve trunks from all patients (*n* = 8). The median mean firing rate (calculated over 10 s) was 0.4 Hz (range 0.0–0.5 Hz), and the median peak firing rate was 2.0 Hz (range 1.0–4.0 Hz). By contrast, spontaneous firing was recorded from only 3 of the 30 colonic nerves studied in 3 of 10 patients. Spontaneous colonic nerve activity was noted to be less intense than rectal nerves, with a median mean firing rate of 0.14 Hz (calculated over 10 s; range 0.14–1.12 Hz) and a median peak firing rate of 1.00 Hz (range 1.00–4.00 Hz).

#### Nerve responses to mechanical stimulation.

Typical responses to mechanical probing of a mechanosensitive hot spot are shown in Fig. 4, *B* and *C*, with reproducible firing on repeated probing, even after several minutes. Hot spots were typically punctate, and recorded activity often appeared to be multiunit rather than single unit, reflecting firing by multiple neurons. Of the 28 rectal nerves studied, 18 hot spots were identified in 16 nerves. The median threshold probing force required to elicit nerve activity was 2.0 *g* (range 1.4–6.0 *g*), and firing rates varied with force (i.e., nerve discharges decreased as probing force decreased toward threshold), as shown in Fig. 4*C*. Generally, only one hot spot was identified per nerve; however, in two nerves, two separate hot spots were identified. By contrast, only one hot spot was identified from the 30 colonic nerves trunks recorded, which had a threshold of 60 *g* to elicit firing.

#### Nerve activity following chemical stimulation.

**Fig. 5.**
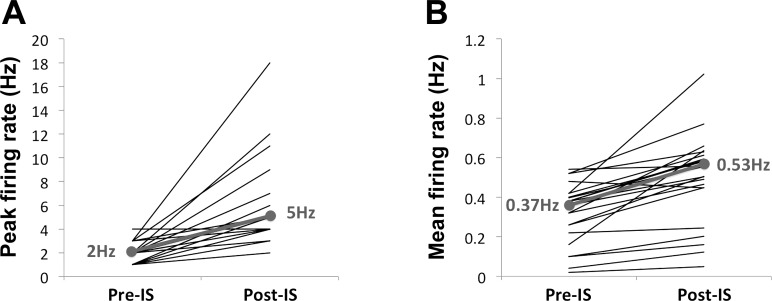
Line graph showing changes in peak (*A*) and mean (*B*) firing rates in rectal nerve activity before/after application of inflammatory soup (IS). The individual lines (black) represent changes in firing rates for individual rectal nerves. The solid gray line in each graph indicates median values of firing rates before and after chemical stimulation, with median values indicated in gray text. The changes in firing rates before and after chemical stimulation were all statistically significant (*P* < 0.001).

**Fig. 6.**
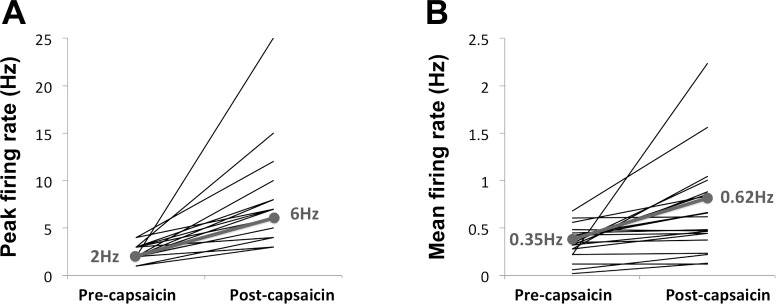
Line graph showing changes in peak (*A*) and mean (*B*) firing rates in rectal nerve activity before/after capsaicin. The individual lines (black) represent changes in firing rates for individual rectal nerves. The solid gray line in each graph indicates median values of firing rates before and after chemical stimulation, with median values stated in gray. The changes in firing rates before and after chemical stimulation were all statistically significant (*P* < 0.001).

In many nerves studied, chemical stimulation (either by application of an IS or capsaicin) resulted in an increase in firing. Responses of rectal nerves to chemical stimulation are presented quantitatively in Figs. 5 and 6. Of the rectal nerves with spontaneous firing (24 nerve trunks), application of IS resulted in a significant increase in mean neuronal firing from 0.37 Hz (median) (range 0.02–0.54 Hz) to 0.53 Hz (range 0.05–1.02 Hz); *P* < 0.001. In addition, peak firing rates increased from 2.00 Hz (range 1.00–4.00 Hz) to 5.00 Hz (range 2.00–18.00 Hz); *P* < 0.001 (Fig. 5). In rectal nerves in which spontaneous activity was not present, application of IS did not induce any discernible nerve activity.

Application of capsaicin consistently evoked a significant increase in nerve activity in rectal nerves in which spontaneous activity was initially recorded (*n* = 24). Specifically, mean firing rate increased from 0.35 Hz (median) (range 0.02–0.68 Hz) to 0.62 Hz (range 0.12–2.23 Hz) (*P* < 0.001), and peak firing rates increased from 2.00 Hz (range 1.00–4.00 Hz) before capsaicin to 6.00 Hz (range 3.00–25.00 Hz) after capsaicin (*P* < 0.001). Application of capsaicin did not induce firing in rectal nerves in which spontaneous activity was not present.

#### Colonic nerve activity following chemical stimulation.

In the three colonic nerves in which spontaneous activity was recorded, application of IS increased mean neuronal firing from 0.14 Hz (median) (range 0.14–1.12 Hz) to 0.19 Hz (range 0.13–1.49 Hz). Peak firing increased from 1.00 Hz (range 1.00–4.00 Hz) to 3.00 Hz (range 2.00–8.00 Hz). The significance of this observation is difficult to interpret given the small number of observations. In one case, application of IS evoked firing (peak firing rate of 2.00 Hz) in a colonic nerve that lacked spontaneous firing. Capsaicin also increased mean firing rate from 0.15 Hz to 0.23 Hz (*n* = 3). Peak discharge rate increased from 2.00 to 9.00 Hz in one of the three colonic nerves in which spontaneous activity was initially recorded; in the other two colonic nerves, there was no increase in peak neuronal firing rate. As observed with IS, capsaicin evoked nerve activity in one colonic nerve trunk that had previously had no spontaneous firing (a different trunk to that activated by IS).

#### Mechanosensitivity to von Frey hairs after IS.

Overall, there was a significant decrease in the mechanosensitivity thresholds during von Frey hair probing following chemical stimulation with IS [median pre-IS 2.0 *g* (range 1.4–6.0 *g*), median post-IS 1.4 *g* (range 0.4–6.0 *g*); *P* = 0.011]. More specifically, the threshold was decreased in eight of the 18 hot spots identified in rectal tissue following chemical stimulation with IS [median pre-IS threshold: 1.7 *g* (range 1.4–2.0 *g*); median post-IS: 1.0 *g* (range 0.4–1.4 *g*)]. Additionally, three additional hot spots were identified on probing following IS application that were not identified before chemical stimulation (median threshold post-IS 1.4 *g*). In the one colonic nerve where a hot spot was identified, application of IS did not alter the threshold force of probing required to elicit nerve activity.

#### Electrical volley.

A random sample (*n* = 6) of colonic nerves that lacked both spontaneous and chemically stimulated nerve activity was investigated using electrical stimulation. Suprathreshold electrical stimulation applied to the surface of the preparation evoked compound action potentials at small punctate sites. Typically, as the electrode was moved away from the center of a stimulation site, the electrical threshold increased abruptly. Conduction velocities calculated from compound action potentials were relatively consistent (mean 1.1 m/s; range 0.8–1.5 m/s), suggesting the presence of axons conducting in the C-fiber range.

## DISCUSSION

This study is the first to develop an in vitro model to successfully record afferent activity from nerves supplying the human rectum and thus functionally demonstrate human rectal innervation via extrinsic nerves. There were demonstrable differences in the sensory innervation between the rectum and colon with rectal afferents being more mechanically and chemically sensitive than colonic afferents. Much of our present understanding of the electrophysiology of rectal afferent nerves was based on findings from animal studies with tracing and immunocytochemical studies revealing the distribution of afferent fibers and their terminals within the gut wall (15, 21). Indeed, elegant in vitro studies of mouse colon and rectum have provided a sophisticated description and electrophysiological classification of visceral afferent fibers, with those responding to *1*) contraction and physiological distension (<20 mmHg) being classified as muscular, *2*) fine mucosal stimulation (e.g., stroking) being referred to as mucosal, *3*) noxious levels of distension (>40 mmHg) being referred to as serosal and mesenteric, and *4*) both tactile and distension stimuli being referred to as muscular-mucosal (1, 6). Furthermore, endings of specialized low-threshold endings of extrinsic afferent neurons in the rectum in the guinea pig have been identified (31, 32, 34). These rIGLEs are located exclusively within the myenteric ganglia and consist of flattened leaflet-like endings, arising from branching axons, and are the mechanotransduction sites of specialized sacral mechanoreceptors (32).

Although focus on human tissue studies has increased in the last decade in neurogastroenterology, few studies have successfully recorded from human extrinsic nerves conveying sensory information along the brain gut axis (43). In the 5 yr since afferent nerve activity was first recorded from the colon and appendix in humans (26, 35), the same two laboratories have extended their preliminary work to identify functionally distinct subpopulations of human visceral afferents supplying different parts of the lower gastrointestinal tract (including the ileum). However, much of the focus was still placed on colonic tissue in these studies (33, 48), and thus no studies have specifically sought to record and characterize activity from human rectal afferents in noninflamed states. Consequently, that was the aim of the present study with the additional goal of a direct comparison between afferents innervating the human rectum and colon using identical protocols. Spontaneous activity was readily recorded from rectal nerves but less so from colonic nerves, and mechanosensitive hot spots were also more abundant in rectal nerve recordings and had lower median thresholds than for colonic nerves, which were rarely responsive to this form of mechanical stimulation. Notably, most recorded activity was multiunit, reflecting firing by multiple sensory neurons, as with many of the recordings made in previous human afferent studies (26, 35). This is in contrast to recordings made in animal studies in which single units can often be discriminated. This difference probably reflects the anatomical differences encountered in human bowel (e.g., increased mural thickness) with a possible more complex three-dimensional arrangement of nerve endings, increased mesenteric adipose and connective tissue (as evident in Fig. 2), and the greater difficulty of dissection of extrinsic nerve trunks. It remains to be seen whether single-unit recordings can be routinely made with refinements in tissue dissection protocols; this warrants evaluation in future studies.

The sparse distribution of colonic hot spots identified in the present study is comparable to the two previous reports, which both reported few mechanosensitive sites (2/9 and 4/27) in colonic preparations (26, 35). Furthermore, differences in nerve activity between rectum and colon corroborate well with previous animal studies (6, 32). In the guinea pig colorectum, fewer colonic nerve units were found to be low-threshold mechanoreceptors than rectal nerve units, consistent with the findings of the present study, and the few colonic nerve units that could be activated by von Frey hair stimulation had substantially higher force thresholds (32). Furthermore, anterograde labeling of colonic nerve and rectal nerve trunks revealed substantially fewer rIGLEs filled per colonic nerve compared with rectal nerves in the guinea pig distal bowel (34). All aspects of our experimental protocol, such as tissue preparation, nerve dissection, and degree of tissue stretch applied, were standardized between rectal and colonic tissue. Additionally, differences between rectal and colonic afferents were unlikely to be due to damage by the dissection because compound action potentials could be evoked by focal electrical stimuli in six out of six colonic nerve trunks, demonstrating that the neural connections with the preparation were intact. The resulting conduction velocities recorded were in the range expected of C-afferent fibers.

This study also used the following two algogenic stimuli to examine neuronal responses to chemical stimuli: *1*) an IS containing a combination of chemical mediators released from inflamed tissue and *2*) a naturally occurring ligand of the transient receptor potential vanilloid 1 (TRPV1), capsaicin. Both caused significant increases in multiunit firing frequency with increased activation of spontaneously active units as well as recruitment of previously “silent” units. The sensitivity to capsaicin confirms that many human visceral afferents (especially in rectal nerves) express TRPV1 receptors, in keeping with the finding from of a previous immunohistochemical study (12). The roles of TRP ion channels in visceral sensation (2) and nociception (4) have been well investigated and described in small animals. For example, treatment with ruthenium red, a TRP channel blocker, reduced distension responses in one study of guinea pig colon afferents (49), whereas TRPV1 (TRPV1−/−), TRPV4 (TRPV4−/−), and TRPA1 knockout mice show markedly attenuated behavioral and afferent responses to colon distension (5, 7, 27, 40, 41). Additionally, TRPV4 has been described to make a specific and major contribution to high-threshold mechanosensory afferent function (7) and as such is the only nociceptor-specific TRP channel as yet identified (2). It is interesting to note that, although the present study revealed a robust response to both IS and capsaicin, the increases in firing rates were smaller than a previous study of human colon following capsaicin and IS (35). It is possible that discarding the mucosa before recording may have removed some classes of endings that were sensitive to these agents; the mucosa was left intact in the previous studies (26, 35).

Sensitization of visceral afferents was demonstrated in the present study by a decrease in thresholds to von Frey hair probing following exposure to IS. Furthermore, three new hot spots were identified after IS, suggesting that previously silent afferents had been activated. Sensitization of visceral afferents by inflammatory mediators has previously been documented in animal studies (19, 47), with one study demonstrating a decrease in electrical stimulus thresholds following chemical stimulation (19). Furthermore, mechanically insensitive afferents (so-called silent afferents) may also be activated by chemical stimulation in small animals (3).

The observed differences in mechanosensitivity between rectal and colonic axons may reflect physiological differences in function between the two regions. Specifically, rectal distension typically elicits different graded rectal sensations, the nature of which qualitatively changes as the distension volume (or pressure) increases from an initial awareness, followed by a desire to defecate, and lastly an intense, irrepressible urge to defecate (18, 37, 45). In contrast, colonic sensation that is less graded manifests as visceral pain during high-pressure distension (24).

This study was limited by the technical and logistical difficulties encountered with human tissue samples. Patients were heterogeneous in age, sex, and comorbidities and were undergoing surgery for organic disease (predominantly cancer) with the theoretic possibility that the tissue was not entirely “normal” although all tissue specimens were procured from the resection margin as far away from the disease process as practical. Furthermore, specimens were probably subject to a degree of ischemia before recording; this was minimized but could not be completely avoided. It was also unrealistic to study rectal and colonic tissues from the same patient (so comparisons were unpaired). Finally, the protocol involved removal of the bowel mucosa, so some classes of mucosally projecting afferents were unlikely to be recorded. In addition, the variable signal-to-noise ratio made single-unit recordings difficult. This meant that it has not been possible to develop an electrophysiological classification scheme for human visceral afferent fibers. Previously, animal studies have revealed that splanchnic and pelvic pathways contain distinct populations of mechanosensitive afferents; the proportions, receptive field distributions, and response properties differed greatly between the regions (6). This was not a realistic aim for the present study, but it is likely that future studies will further characterize and electrophysiologically classify rectal afferent fibers as achieved in animals.

For the first time, afferent innervation of the human rectum via extrinsic nerves has been functionally demonstrated using in vitro electrophysiological techniques. This study has also demonstrated differences in the sensory innervation of the rectum compared with the colon, consistent with the physiological differences between these parts of the gastrointestinal tract. Preparations from laboratory animals have been used to develop disease models, offering insight into the mechanisms of action for potential pharmacological treatments (10). Nevertheless, a number of novel drug candidates have failed to prove effective in clinical practice despite robust mechanistic data from animals (20, 25, 26, 43). The feasibility of an in vitro model of human rectal sensory nerves may allow examination of the effects of novel therapeutic agents on visceral afferents, avoiding the issue of interspecies differences and providing a valuable supplement to animal models.

## GRANTS

Dr. Kheng-Seong Ng is supported by an NHMRC Postgraduate Research Scholarship and a Royal Australian College of Surgeons (RACS) Foundation of Surgery Research Scholarship.

## DISCLOSURES

No conflicts of interest, financial or otherwise, are declared by the authors.

## AUTHOR CONTRIBUTIONS

K.-S.N. and D.A.M. performed experiments; K.-S.N. and D.A.M. analyzed data; K.-S.N., D.A.M., and M.A.G. interpreted results of experiments; K.-S.N. prepared figures; K.-S.N. drafted manuscript; K.-S.N., S.B., and M.A.G. edited and revised manuscript; K.-S.N., S.B., N.M.-A., D.A.M., and M.A.G. approved final version of manuscript.
